# Exercise Increases Bone in SEIPIN Deficient Lipodystrophy, Despite Low Marrow Adiposity

**DOI:** 10.3389/fendo.2021.782194

**Published:** 2022-01-25

**Authors:** Cody McGrath, Sarah E. Little-Letsinger, Jeyantt Srinivas Sankaran, Buer Sen, Zhihui Xie, Martin A. Styner, Xiaopeng Zong, Weiqin Chen, Janet Rubin, Eric L. Klett, Rosalind A. Coleman, Maya Styner

**Affiliations:** ^1^ Department of Medicine, Division of Endocrinology & Metabolism, School of Medicine, University of North Carolina at Chapel Hill, Chapel Hill, NC, United States; ^2^ Department of Psychiatry, School of Medicine, University of North Carolina at Chapel Hill, Chapel Hill, NC, United States; ^3^ Department of Computer Science, University of North Carolina at Chapel Hill, Chapel Hill, NC, United States; ^4^ Biomedical Research Imaging Center, University of North Carolina at Chapel Hill, Chapel Hill, NC, United States; ^5^ Department of Physiology, Medical College of Georgia at Augusta University, Augusta, GA, United States; ^6^ North Carolina Diabetes Research Center (NCDRC), Chapel Hill, NC, United States; ^7^ Department of Nutrition, Gillings School of Global Public Health, UNC, Chapel Hill, NC, United States

**Keywords:** exercise, endocrinology and metabolism, congenital lipodystrophy, bone, SEIPIN, BSCL2, anabolism, bone marrow adipose tissue (BMAT)

## Abstract

Exercise, typically beneficial for skeletal health, has not yet been studied in lipodystrophy, a condition characterized by paucity of white adipose tissue, with eventual diabetes, and steatosis. We applied a mouse model of global deficiency of Bscl2 (SEIPIN), required for lipid droplet formation. Male twelve-week-old B6 knockouts (KO) and wild type (WT) littermates were assigned six-weeks of voluntary, running exercise (E) versus non-exercise (N=5-8). KO weighed 14% less than WT (p=0.01) and exhibited an absence of epididymal adipose tissue; KO liver Plin1 via qPCR was 9-fold that of WT (p=0.04), consistent with steatosis. Bone marrow adipose tissue (BMAT), unlike white adipose, was measurable, although 40.5% lower in KO vs WT (p=0.0003) via 9.4T MRI/advanced image analysis. SEIPIN ablation’s most notable effect marrow adiposity was in the proximal femoral diaphysis (-56% KO vs WT, p=0.005), with relative preservation in KO-distal-femur. Bone via μCT was preserved in SEIPIN KO, though some quality parameters were attenuated. Running distance, speed, and time were comparable in KO and WT. Exercise reduced weight (-24% WT-E vs WT p<0.001) but not in KO. Notably, exercise increased trabecular BV/TV in both (+31%, KO-E vs KO, p=0.004; +14%, WT-E vs WT, p=0.006). The presence and distribution of BMAT in SEIPIN KO, though lower than WT, is unexpected and points to a uniqueness of this depot. That trabecular bone increases were achievable in both KO and WT, despite a difference in BMAT quantity/distribution, points to potential metabolic flexibility during exercise-induced skeletal anabolism.

## Introduction

Exercise -induced skeletal anabolism is a vital physiologic process, which in addition to calcium, vitamin D, and hormonal stimuli, necessitates substrate energy, fueling osteoblasts as they lay new bone (1). An array of cells orchestrate this bone-building response to mechanical loading (2–11), including osteocytes, osteoclasts, endothelial cells, and their progenitors. Osteoblasts, while able to use various substrates (12) prefer energy-rich fatty acids (13). Bone marrow adipose tissue (BMAT) likely serves as the energy depot in exercising, non-calorically restricted mice (14–16) but it is unknown if metabolic flexibility permits use of other substrates to reproducibly build bone. Here we asked whether exercise-induced bone is achievable in the setting of a mouse model of congenital generalized lipodystrophy (CGL), characterized by a near absence of peripheral white adipose tissue stores.

Human studies show a varied effect of partial and generalized congenital lipodystrophies on bone, which might be due to disease-specific mutations. An increase in bone quantity has been noted, along with advanced skeletal age; yet, there is also evidence of skeletal ‘harm’, in the form of cystic or osteolytic bone lesions (17–19). Lima et al. shows a higher bone mass in trabecular sites, contrasted with a reduction in the distal radius, a cortical site (20). A comprehensive radiologic analysis of the skeleton in 3 CGL patients (21) reports cystic bone lesions, multiple long bone defects, reduced BMAT, periarticular hyperemia, as well as increased activity via triple phase scintigraphy in some skeletal sites. In some (mutations Bscl2, Agpat2) there is a reduction in marrow fat via MRS (22–24); while in others (mutations CAV1, LMNA) a preservation of BMAT is noted (25, 26). Mouse models like A-ZIP fat-less (27), Cav1 ^(-/-)^ (28), transgenics applying diphtheria toxin to ablate adipocytes (29) or PPARγ^(+/-)^ (30), reveal increased bone quantity or osteosclerosis. The precise effect of CGL generally, and specifically that of Bscl2/SEIPIN deficiency on bone, remains essentially unknown. Defined by an inability to store triglycerides in adipocytes, lipodystrophy results in diabetes, hypertriglyceridemia, heart disease, and intellectual disability (31–33). In humans, the most clinically severe CGL arises due to loss of function of Bscl2 (34), encoding SEIPIN, which packages lipid and protein cargo into maturing droplets necessary for adipogenesis (34–37). We thus hypothesized that in a mouse model of SEIPIN deficiency, attenuated BMAT storage or function might alter exercise -induced bone formation.

Due to numerically limited human studies of CGL, animal studies are vital in the interrogation of BMAT and bone-response to exercise. Methods to quantify and visualize BMAT distribution in rodents are also unparalleled in human trials (15). We asked if adiposity in the marrow is measurable in lipodystrophic mice, and whether exercise regulates bone, when adipose stores are scant. We were surprised to find a quantifiable marrow adipose tissue (BMAT) depot in the SEIPIN KO mice by histomorphometry and MRI. While SEIPIN KO BMAT was present, it was lower than WT, particularly in the proximal femur, with relative preservation of BMAT in the distal femur. The presence of BMAT in SEIPIN KO contrasted with an absence of white adipose tissue. We also discovered that exercise-induced trabecular bone was robust in SEIPIN KO, even without exercise-induced attenuation of BMAT, highlighting a possibility that SEIPIN KO rely on an alternative substrate to fuel skeletal anabolism.

## Materials and Methods

### Animals, Diet, and Exercise Intervention

Procedures and ethical guidelines of the University of North Carolina’s Institutional Animal Care and Use Committee (IACUC) were adhered throughout the study. Male, twelve-week-old, wild-type (WT) and global SEIPIN knock-out (KO) mice (laboratory of Weiqin Chen) were randomized to running or sedentary groups as available due to breeding. Global SEIPIN KO mice were generated as previously described (38, 39). Briefly, Bscl2 exon 3 germ-line deletion was accomplished by crossing mice with loxP Bscl2 allele to mice expressing Cre recombinase driven by germ (oocyte) specific promoter (Zp3-cre, Jax #003394). Screening of progeny via PCR for loss of the Bscl2 exon 3 was performed. After the Bscl2-null allele was generated, the strain was crossed with C57BL/6 for 3 generations and inbreed with heterozygotes to obtain homozygotes. The Bscl2-nulls were backcrossed at least 6 times prior to initiation of these experiments. C57BL/6 mice of this age were selected as BMAT and exercise response has not previously been quantified in this sample. Males were selected due to an abundance of prior metabolic data in male 12-week-old SEIPIN KO (39). Mice were housed in controlled light (12-hour light/12-hour dark), temperature (21-22 degrees Celsius), and humidity (range 30-70%) conditions, with ad libitum access to food and water, and acclimated for 5 days prior to initiation of experiments. Mice were randomly assigned to the following experimental groups for 6 weeks: non-exercising WT (WT, n=5) (2), exercising WT (WT-E, n=6), (3) non-exercising KO (KO, n=8), and (4) exercising KO (KO-E, n=6). For MRI analyses, WT-E group was excluded due to an insufficient number of specimens. Running intervention length did not differ between groups. Mice were 18 weeks of age at harvest; age at harvest did not differ between groups. Mice were individually housed for the duration of the experiment to track daily wheel running. Exercise groups were provided access to running wheels as previously described (5, 40, 41). Wheel use was monitored using a Mity 8 Cyclometer (CC-MT400, Cat Eye, Osaka, Japan). All mice were fed a standard 10% fat diet (#D12450H, Research Diets, New Jersey, USA) for the duration of the experiment. Body weights were measured weekly.

### 3D Quantification and Imaging of Bone Marrow Adipose Tissue

Quantification of BMAT was performed via high-resolution 9.4T MRI with advanced image analysis for 3D volumetric BMAT analysis, a method previously validated against both osmium-stained-μCT with advanced image analysis, as well as histomorphometry (see **Expanded Supplemental Methods, Supplementary Figure 1**) (14, 15). Mechanical loading or weight-bearing exercise in rodents has been shown to affect hind limbs, e.g., tibia and femur, but also other sites (42–44). Progenitor populations in the femur (10) are more exercise-responsive, though both have strong response and are therefore preferred for exercise experiments, as compared with forelimbs (10). Briefly, femurs were analyzed with a 9.4T horizontal small-bore MRI scanner to quantify BMAT volumetrically (14, 15). Water and fat maps were obtained with a 2-dimensional RARE imaging sequence with the following parameters: RARE factor = 4, TE = 28 ms, TR = 4000 ms, number of averages = 4, number of slices = 24, slice thickness = 0.5 mm, in-plane resolution = 100×100 μm2, matrix size = 130×130. Utilizing the fact that the fat and water protons have an NMR frequency separation of 3.5 ppm, a Gaussian-shaped 90-degree saturation pulse with a width of 2 ms was applied preceding the RARE sequence to suppress the fat or water signal while the other signal remained unaffected. Fat and water images were acquired by setting the saturation pulse frequency to be the same as the water and fat frequencies, respectively.

In our processing workflow, we manually subdivided the full images containing all 10 samples into individual images for each bone. Then, we employed water images to manually outline femoral bone masks using Insight ITK-SNAP (open-access www.itksnap.org) (45). Using these bone masks, interior bone regions were masked from other image parts in both water and fat maps. Next, we established a common, study-specific reference space by computing an unbiased average image (46) from masked water maps using the ANTs registration software (47). All individual water and fat maps were then propagated into the common space, where voxel-wise correspondence allows direct comparison of intensities. Average fat maps for each experimental group were computed in the common space and superimposed on the common, average water image for visualization of group fat maps as in **Figure 2A**. Fat map intensities were represented with a colored heat map in 3DSlicer for visualization (open-access www.slicer.org) (45, 48). 3D Slicer is a platform distributed under a BSD-style open-source license that is broadly compatible with the Open-Source Definition by The Open-Source Initiative and contains no restrictions on legal uses of the software. For BMAT quantification, we created a regional label map of the femur, excluding cortical bone regions, with regions for the epiphysis, metaphysis, and diaphysis. Intensity weighted volume of BMAT was then quantified via regional fat histograms.

### Bone Marrow Adipocyte Quantification via Histomorphometry

Fixed and decalcified femurs were embedded in paraffin, sectioned at 5 µm, stained with hematoxylin (MHS16, Sigma-Aldrich, St. Louis, MO, USA) (14, 49). Imaging on an Olympus X81 at 4x and 40x.The 40x images were obtained at the distal femoral metaphysis, where lipid content is maximal. ImageJ was used to isolate adipocytes within 40x images and to quantify adipocyte size as previously described (50). Globular maxima were removed manually to isolate defined adipocytes. The “Analyze Particles” function was used to outline cells and calculate area in pixels. A lower limit of 1000 pixels was applied to N=2-3 mice/group, with a minimum of 5 distinct histologic regions per section, as well as a minimum of 3 sections per animal. A minimum of 20 adipocytes were analyzed for adipocyte area measurement per mouse in the 3 sections. To assess adipocyte number via ImageJ, the marrow cavity at the distal metaphysis was imaged at a magnification of 4x and adipocytes were manually counted. Adipocyte number was then normalized to the total metaphyseal area assessed to obtain adipocyte number per square (#/mm2). Presence of adipocytes in the femur of B6 mice of similar age, via H&E as well as perilipin 1 immunohistochemistry are shown in **Supplementary Figure 1** (**Supplementary 1A–C** distal femoral metaphysis, **Supplementary 1D** proximal femoral diaphysis).

### Bone Microarchitecture and Biomechanical Testing

Bone microarchitecture parameters of the proximal tibia metaphysis and mid diaphysis were quantified ex-vivo via high resolution μCT (Scanco μCT40, UNC- Chapel Hill Biomedical Imaging Research Center, resolution 12 μm; E 55 kVa; I 145 μA) (51, 52). Briefly, the interface of trabecular and cortical surfaces was manually contoured. To resolve the compartments, natural contour was preserved at the endosteum, where the cortical surface was delineated from trabecular struts based on variations in discrete density values (mgHA/ccm). Subsequently, an automated algorithm was applied to quantify bone in each compartment. Parameters analyzed in trabecular bone include: Ratio of the segmented bone volume to the total volume of region of interest (Bone Volume Fraction; BV/TV); Measure of average number of trabeculae per unit length (Trabecular number; Tb.N); Mean thickness of trabeculae, assessed using direct 3D methods (trabecular thickness; Tb.Th); Mean distance between trabeculae, assessed using direct 3D methods (Trabecular separation; Tb.Sp). Parameters in cortical bone include: Total cross sectional area inside the periosteal envelope (Tt.Ar); Cortical bone area (Ct.Ar) calculated from cortical volume divided by number of slices x slice thickness; Cortical area fraction (Ct.Ar/Tt.Ar); Average cortical thickness (Ct.Th).

### TRAP Stain

Femurs were fixed in 10% formalin, decalcified in formic acid (UN3412 Immunocal, StatLab, Texas, USA), paraffin-embedded, and sectioned longitudinally at 5 µm, and mounted on glass slides. Xylene- deparaffinized sections were rehydrated with graded ethanol, rinsed with deionized water, and stained for TRAP by a buffer containing Naphthol-AS-BI-phosphate (70485, Sigma-Aldrich, St. Louis, MO, USA) followed by a buffer containing Sodium Nitrite (237213, Sigma-Aldrich, St. Louis, MO, USA) and Pararosaniline dye (215600, Sigma-Aldrich, St. Louis, MO, USA). Fast Green stain was applied (F7252, Sigma-Aldrich, St. Louis, MO, USA), dehydrated, then exposed to Xylene before mounting with Cytoseal (8312-4, Thermo Fisher Scientific, MA, USA). Images were obtained via an Olympus IX81 and TRAP was quantified using open-source software (50).

### Real-Time qPCR

Whole tibia mRNA was reverse transcribed and analyzed via real-time qPCR as previously described (53–55). Briefly,Ten µL of cDNA from each experimental condition were pooled and diluted 1:10 to 1:10,000 to generate a 5-point standard curve. A non-template control was added to each PCR reaction. Standards and samples were run in duplicate. PCR products were normalized to GAPDH. PCR primer sequences are available in **Supplemental Table 1**.

### Statistical Analysis

Analyses were performed using GraphPad Prism Version 9.1.0 (GraphPad, San Diego, CA, USA). Data sets passed the Shapiro-Wilk normality test. We applied the one-way or two-way analysis of the variance or ANOVA (genotype x exercise) with correction for multiple comparisons via Tukey’s *post-hoc* test. Significance was defined as an α less than 0.05. Adipocyte area was assessed via nested ANOVA. Running parameters (WT-E vs KO-E) were analyzed using an unpaired, two-tailed t-test.

## Results

### SEIPIN Deficient Mice Run Similarly to Wild Type

**Figure 1:**
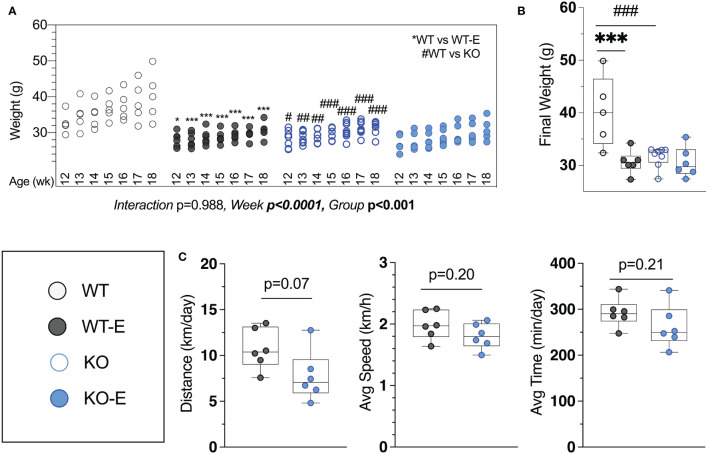
Lipodystrohic SEIPIN KO run similar to WT despite lower body weight. Male SEIPIN KO and WT littermates allocated to voluntary running exercise (E) or non-exercise groups (KO, WT) at twelve weeks for a six-week experimental period (N=5-8). **(A)** Individual body weights throughout and **(B)** at harvest. **(C)** Voluntary wheel running metrics via cyclometer- average daily running distance (km/day), average running speed (km/h), and average daily running duration (min/day) in WT-E and KO-E. Data as individual values on box and whisker plots (min to max). Horizontal lines represent means. Significance via 2-way ANOVA with Tukey's *post hoc* **(A, B)** or unpaired t-test **(C)** Between-group * or ^#^p < 0.05; **or ^##^p < 0.01; ***or ^###^p < 0.001.

To investigate the effect of exercise on bone and BMAT, we employed a lipodystrophic mouse with global ablation of Bscl2, encoding SEIPIN, a protein required for lipid droplet formation. Our prior work showed that these Bscl2 KO mice are hyperglycemic and hyper insulinemic by 10 weeks of age, though this was not quantified in the present cohort (39). Lipodystrophic KO weighed less than WT littermates throughout the experimental period, with a final weight 14% less than WT (p=0.01) (**Figures 1A, B**). Prior studies similarly note reduced weight in KO, though at a younger age (39). Consistent with loss of SEIPIN (39, 56), KO -exercisers and non- exercisers- lacked epididymal fat pads. In terms of other adipose depots, prior work by our team (39) in the same Bscl2-/- mice showed loss or reduction of several depots including subcutaneous, interscapular, perirenal, gonadal WAT, as well as a histological appearance of smaller adipocytes possessing mostly unilocular lipid droplets. Regarding lean mass, prior work in the same Bscl2-/- mice showed an increase in lean mass consistent with lipodystrophy (39). The liver Plin1 via qPCR was 9-fold higher in SEIPIN KO than WT (p=0.04), consistent with probable steatosis (39), which was also supported by gross examination demonstrating enlarged, pale livers (**Figure 6**).

Harvest weights were highest in the WT non-runners (40.3 ± 7.8 g **Figure 1A**). Exercise associated with significantly reduced weight in WT-E vs. WT at each week of the experimental period (**Figure 1A**), consistent with prior work (14, 15, 41) Notably, we found that KO-E weight did not differ significantly from KO, diverging from the exercise-effect on body weight noted in WT. One of the early publications by Seip et al. (56) pointed to hyperphagia in this form of lipodystrophy in humans. Based on this and our prior work in mice (39), increased caloric intake in SEIPIN KO is anticipated. It is possible that caloric intake in SEIPIN KO-E was attenuated as lipodystrophy has been associated with hyperphagia in the literature (39), however as caloric intake was not quantified in our analysis, we cannot firmly state the cause for this.

Daily running parameters were similar in KO-E (7.8 ± 2.7 km/day **Figure 1C**) and WT-E (10.7 ± 2.2 km/day, p=0.07 though trended less). Average running speed (1.81 ± 0.21 km/hr. in KO-E; 2.0 ± 0.2 km/hr. in WT-E, p=0.20) and daily running time were similar between groups (262 min ± 46 min in KO-E; 292 ± 31 min in WT-E vs, p=0.2)

### Distal Femur BMAT Is Present in SEIPIN KO, and Reduced in the Proximal Femur Diaphysis

**Figure 2:**
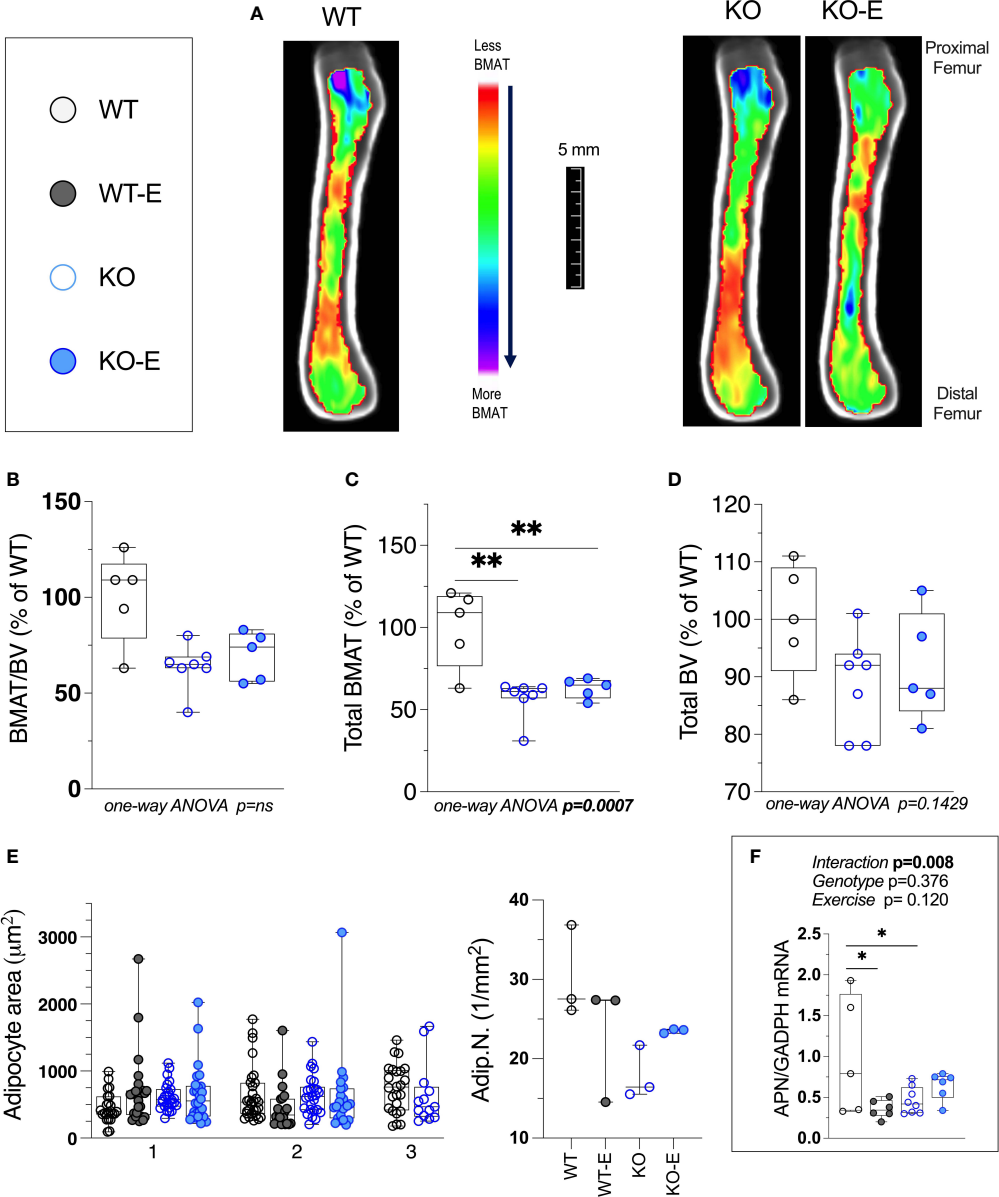
Bone Marrow adipose tissue (BMAT) is extant, but reduced in SEIPIN KO Lipodystrophy. 12-week­ old male Bscl2 ^-/-^ KO allocated to exercise (E) vs non-exercise for 6 wk. compared to WT **(A)** Visualization of BMAT via 9.4 T MRI with advanced image analysis; each image is an average from superimposed individual images in the sagittal plane. Volumetric quantification of BMAT form N=5-7 MRI images is shown in **(A)**. In **(B–D)**, BMAT values are shown as % relative to WT mean. **(B)**: Bone Marrow Adipose Tissue /Bone Volume (BMAT/BV). **(C)**: Total Femoral Bone Marrow Adipose Tissue (BMAT). **(D)**: Bone Volume (BV). Group differences in **(B–D)** were analyzed via one-way ANOVA with Tukey's post-hoc test, **p < 0.01. Due to insufficient number of specimens, WTE group was excluded. **(E)** plot of histomorphometric analysis: adipocyte area (1μm^2^) and number (1/mm^2^), via lmageJ, with X-axis representative of 2-3 experimental animals. For data in **(E)** there were no significant differences between groups by nested one-way ANOVA. **(F)**: Whole tibia adiponectin (APN) mRNA via qPCR (n=5-8 *I* group) via 2-way ANOVA, Tukey's *post hoc*, between-group *p < 0.05. Data as individual values on box and whisker plots (min to max). Horizontal lines represent means.

**Figure 3:**
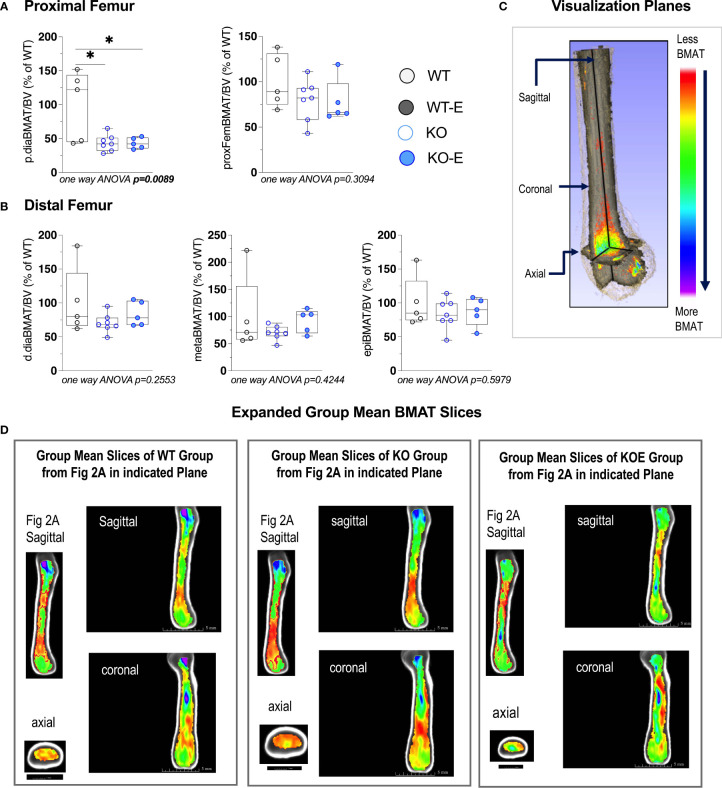
Seipin KO exhibit reduced BMAT in the proximal femoral diaphysis. Twelve-wk.-old male SEIPIN KO allocated to exercise (E) vs non-exercise for 6 weeks, compared to wild type (WT) (n=5-8). BMAT quantified by 9.4T MRI with advanced image analysis to analyze regional distribution of BMAT relative to bone volume (BV). Data expressed as relative% BMAT to WT mean. **(A)** BMAT/BV assessed at the hip and the proximal diaphysis (p.dia). **(B)** BMAT/BV assessed at the distal diaphysis (d.dia), metaphysis (meta), and epiphysis (epi). Analyzed via one-way ANOVA with Tukey's post hoc, *p < 0.05. Data as individual values on box and whisker plots (min to max). Horizontal lines represent means. **(C)** Visualization planes for 9.4T MRI inboth **Figures 2** and **3**. **(D)** Extended group mean slices obtained at indicated planes.

Next we turned to investigate BMAT via 3-dimentional MRI in the 9.4T scanner, quantifying and localizing femoral BMAT with advanced image analysis (**Figures 2A–D** and **3A–D**) (5, 45–47) (BMAT quantification: **Extended Supplementary Methods, Supplementary Figure 1**) BMAT was visualized via superimposed average group images (**Figure 2A**). It is notable that BMAT was extant and quantifiable in SEIPIN KO, though measuring 40.5% less than WT (p=0.003) SEIPIN ablation resulted in a significant reduction of BMAT in the hip, specifically the proximal diaphysis (-56% KO vs WT, p=0.005, **Figure 3A**), with relative preservation of BMAT in the KO-distal-femur (**Figure 2B**). Prior work demonstrated that exercise associates with reduced BMAT in WT animals fed ad-libitum (14, 15, 40, 41). Due to insufficient number of specimens (absent running data in one animal and poor MR image quality in another). WT-E group was excluded from this analysis, but the group was not required to test our hypotheses (14, 41). The WT-E group was included in other analyses such as bone microarchitecture, qPCR and histomorphometry. Histomorphometric quantification of marrow adipocytes via hematoxylin staining showed similar average adipocyte number and size across the groups (**Figure 2E**). The high-resolution MRI sequence for volumetric BMAT quantification correlates well with osmium stained-μCT analyses, as well as histomorphometry (15).While it is valuable to show histological support for 3-dimensional, volumetric BMAT via high resolution MRI, it is the MRI data that is most conclusive representing higher volume throughout the entire bone; thus, our conclusions were largely based on MRI quantification of BMAT. Whole bone adiponectin mRNA was 53% lower in KO vs. WT (**Figure 2F**, p=0.039) and 64% lower in WT-E vs WT (p=0.018). Whole tibia mRNA of white and brown adipose tissue markers, as well as bone markers, did not significantly vary between groups (**Figure 6**). In sum, the presence of BMAT in SEIPIN KO was notable, and the fact that BMAT was more proximally distributed in the femur, than in WT based on MRI quantification (**Figures 3A–D**).

### Increased Bone in Exercisers, Both WT and Lipodystrophic SEIPIN KO

**Figure 4:**
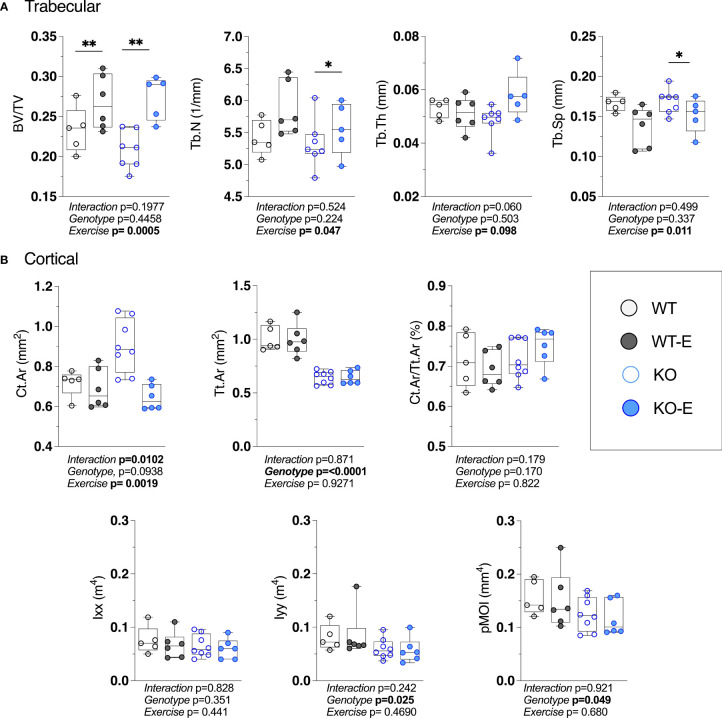
Higher bone quantity in exercising WT and lipodystrophic SEIPIN KO mice. Twelve-week-old male SEIPIN KO and WT allocated to exercise (E) vs non-exercise for 6 weeks (n=S-8). **(A)** Trabecular bone microarchitecture (BV/TV, Tb.N, Tb.Th, Tb.Sp) assessed at the proximal tibial metaphysis via 1JCT. **(B)** Cortical geometry (Ct.Ar, Tt.Ar, Ct.Ar/Tt.Ar) and biomechanical measures (lxx, lyy, pMOI) assessed at the mid-tibial diaphysis. Data plotted as individual values with means represented by horizontal lines. Significance assessed via 2- way ANOVA, Tukey's *post hoc.* Significance for between-group comparisons: *p < O.OS; **p < 0.01. Data as individual values on box and whisker plots (min to max). Horizontal lines represent means.

While in humans there is much to be discovered about the type of exercise that optimally stimulates (57–60) bone, wheel running in rodents reproducibly associates with skeletal anabolism (15, 40, 41). Moreover, as wheel running increases bone alongside a diminution of BMAT (15, 40, 41, 61), we asked whether BMAT, as well as bone quantity/quality, are regulated by exercise SEIPIN deficiency. At the outset, we hypothesized that loss of SEIPIN would lower BMAT, thus lessening exercise-induced skeletal anabolism. To our surprise, despite the lower quantity of BMAT in SEIPIN KO, exercise increased trabecular BV/TV in both groups (+31%, KO-E vs KO, p=0.004; +14%, WT-E vs WT, p=0.006, **Figure 4A**), despite a difference in BMAT quantity/distribution (**Figures 2A, B** and **3A–D**). The between-group comparisons for BV/TV revealed p= 0.9797 for WT-E vs KO-E. Exercise accounted for 43% of the total variance, while genotype alone accounted for only 1.48% of the variance. When combined, genotype plus exercise account for 4.5% of the total variance. Other trabecular parameters such as trabecular thickness and separation were similarly responsive to exercise in both KO and WT groups (**Figure 4A**).

With the cortical parameters, a genotype effect was stronger than that of exercise. Specifically, Tt.Ar was 35% lower in KO vs WT (**Figure 4B**, p<0.0001) accounting for 78% of the variance, a finding that is possibly related to lower body weight in SEIPIN KO. The Ct.Ar was ~ 10% higher in KO (**Figure 4B**) however this did not reach significance (p=0.09).The cortical bone volume fraction (Ct.Ar/Tt.Ar) failed to show an effect of genotype or exercise in our analysis (**Figure 4B**).

**Figure 5:**
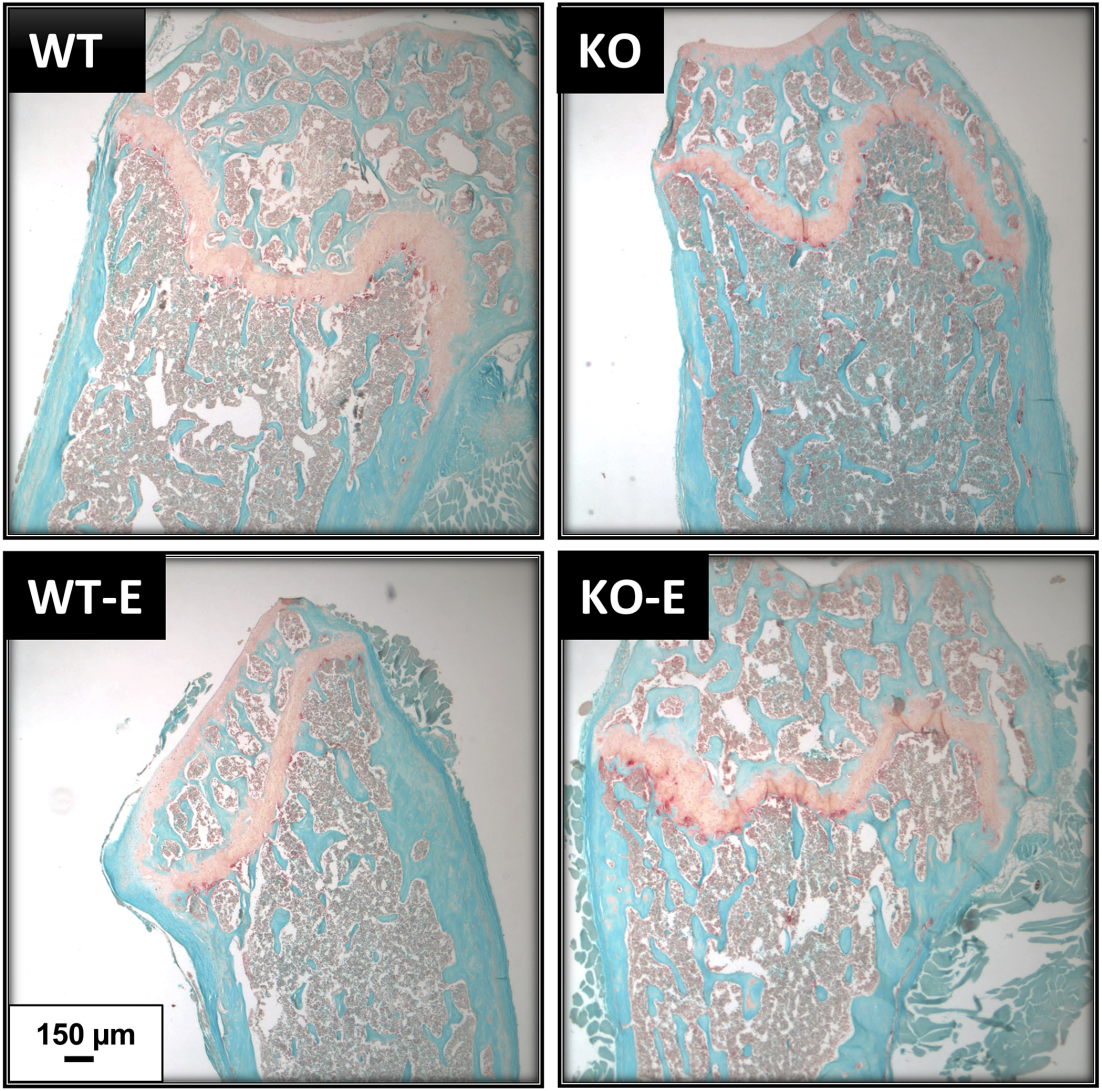
TRAP staining shows similar osteoclast numbers in WT and SEIPIN KO. Twelve-week-old male SEIPIN KO and WT were allocated to exercise (E) vs non-exercise controls for 6 weeks. Representative histologic sections of the distal femur were stained with Fast Green and TRAP, images via Olympus IX81.

Consistent with cortical geometry, polar moment of inertia (pMOI) in SEIPIN KO was reduced by -21% (p=0.049 **Figure 4B**) and the rotational moment of inertia (MOI), an index of resistance to bending, was – 38% in the YY (Iyy) plane vs WT (p=0.025 **Figure 4B**) pointing to possible cortical bone loss. Histomorphometric analysis of TRAP-stained sections (**Figure 5**) showed a similar quantity in the experimental groups.

## Discussion

The skeleton demonstrates an anabolic response to exercise in animal (6, 15, 62–64) and human (60, 65, 66) studies. Despite clinical guidelines recommending lifestyle and exercise intervention (67) as a first-line therapy the effect of mechanical loading or exercise on bone and BMAT, has not been investigated in lipodystrophy. Our preclinical data point to a robust increase in trabecular bone quantity with exercise in lipodystrophic SEIPIN KO mice, despite a scarcity of peripheral adiposity, as well as less BMAT in the proximal femoral diaphysis. These findings, pertinent not only for an improved understanding of a musculoskeletal response to exercise, should prompt additional study of the therapeutic potential of non-pharmacologic, exercise-based therapies for lipodystrophy.

We previously suggested that marrow adipose stores provide metabolic substrates for exercise-induced bone formation (15, 40, 41). It remains unclear if bone cells and their precursors harbor metabolic flexibility, as related tissues like skeletal muscle and cardiomyocytes preferentially rely on fatty acid β-oxidation for ATP during exercise (68, 69). Metabolic flexibility is discussed in reference to skeletal cells known to require fatty acid oxidation (70) though many cells in the niche participate in bone turnover and could be impacted by substrate availability. The reduced peripheral white adipose stores in SEIPIN KO means that they have a reduced capacity to buffer fatty acids from exogenous and endogenous sources and serve as a storage depot, thought to result in metabolic inflexibility that requires a constant supply of metabolic substrates (71). Few studies compare CGL patients with healthy, age-matched controls, and thus it is unknown what are the expected differences in body weight with and without exercise. Percent change in body weights were as expected in WT exercisers compared to control (72). Remarkably, exercise in SEIPIN KO did not result in weight loss, which might be due to known hyperphagia in CGL, thus mitigating the metabolic inflexibility, though this cannot be stated with certainty since caloric intake was not quantified in our study. Thus, our current study with a robust exercise-induced bone in SEIPIN KO means that BMAT might have no role or potentially a different role in skeletal anabolism of SEIPIN KO. Abundant *in vitro* data suggests mechanical signals, mediated by Wnt/β-catenin, cytoskeletal elements, as well as epigenetics -e.g., EZH2- (73) serve to bias mesenchymal stem cells away from the adipocyte lineage, thereby promoting bone; yet these questions remain to be definitively answered *in vivo*. Many of these mechanisms are likely operating in concert with metabolism to achieve exercise-induced bone formation. Future work is needed to improve our understanding of how exercise impacts bone health and metabolic health in lipodystrophy.

Our finding of quantifiable but lower quantity of BMAT in SEIPIN KO is new and provides a systematic quantification of this depot in a mouse model of severe generalized congenital lipodystrophy. In humans, CGL via SEIPIN deficiency is characterized by an absence of white adipose tissue stores (31, 32, 74, 75). Marrow Adipose Tissue in lipodystrophy has not been sufficiently quantified due to the rarity of this disorder (17, 18, 20, 21, 76); though it has been investigated in rodents in other less severe forms of lipodystrophy (PPARγ ^+/-^ or Cav1^-/-^) (77). A study of adipocyte-specific deletion of Bscl2, rather than global deletion, showed preservation of vertebral BMAT with a relative loss of tibial (proximal and distal) BMAT, though these animals do not exhibit metabolic dysfunction as noted in global SEIPIN deletion (71). Prior case reports measured BMAT via biopsy or MRI however the small subject number in these precludes a definitive answer (23–25). Interestingly, the prior human studies had mixed reports on preservation of bone marrow adipose across CGL causal mutations (23–25). This study demonstrated that global ablation of SEIPIN in mice associated with reduced marrow adiposity in the proximal femur- and relative preservation in the distal femur. This finding of preservation at the distal end of a mechanically sensitive long bone and the reduction of BMAT proximally, might be viewed as consistent with the prior literature in both human and animal studies which was mixed and reinforces the need for quantitative volumetric investigations of BMAT that might clarify the physiologic role of the distal vs the proximal femoral BMAT depot.

Prior studies suggested an increase in bone mass in CGL along with cystic bony lesions. Here, SEIPIN KO mice had similar trabecular bone metrics compared to WT, while cortical parameters demonstrated a negative effect of genotype on cortical bone geometry. Our data fits with clinical reports of bone parameters in CGL patients, assessed via DXA, showing increased density primarily at trabecular sites, whereas cortical sites, like the radial diaphysis, is decreased (20).

**Figure 6:**
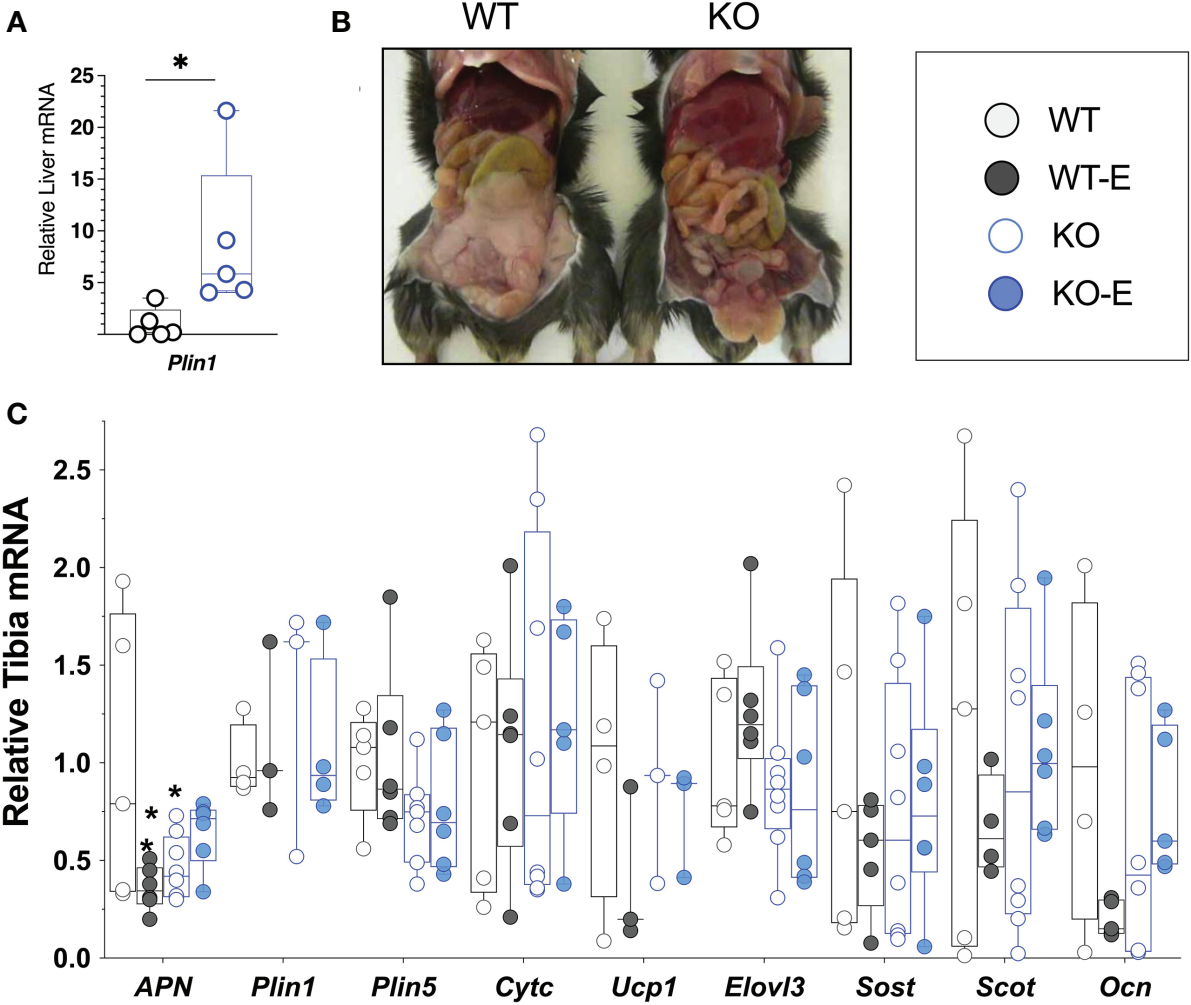
SEIPIN global KO demonstrate higher liver *Plin1* expression, consistent with lipodystrophy. **(A)** Liver mRNA (N=5) via qPCR shows relative expression of *Plin1.* **(B)**, Representative photo of KO and WT demonstrating lipodystrophic liver phenotype in KO. **(C)** Whole Tibia mRNA (N=4-8) via qPCR for metabolism, bone markers. Box and whisker plot, min to max with means. Between group differences as *p < 0.05 vs. WT.

Several adipokines are known to play a role in whole body metabolism and possibly in the regulation of BMAT (78). Though not assessed here, leptin plays a key role in the regulation of bone mass (79) and its deficiency has been proposed to be causal in driving the bone phenotype in lipodystrophy (80). However, despite a beneficial therapeutic effect on metabolism in CGL patients, leptin therapy fails to attenuate bone mass (23). Adiponectin is associated with fat mass, particularly in exercise-induced weight loss (81). CGL lipodystrophic patients exhibit decreased serum levels of adiponectin, consistent with global reduction of adipocytes (82). In bone, we found significantly lower adiponectin mRNA in SEIPIN KO which might be due to lower BMAT; serum adiponectin levels were not assessed. Prior work suggested Bscl2 may play a role in brown adipose tissue function (83), however BAT-specific Bscl2 deletion showed Bscl2 is not required for brown adipogenesis, but rather plays a cell-autonomous role in mediating BAT development and function (84, 85). Marrow adipocytes in our global Bscl2 KO and WT sections were unilocular and similar between KO and WT, though locularity and mRNA analyses (**Figure 6**) are insufficient to exclude brown/beige properties and require additional investigations (UCP1 staining, or ultrastructural as well as bioenergetic analyses of mitochondria (86, 87), are important for future studies. No difference in bone mRNA for sclerostin and osteocalcin was found between groups, even with exercise-induced bone formation, in line with prior work (14). Studies that showed an attenuation of sclerostin/osteocalcin in BSCL (88) measured these in the serum, whereas our study investigated these solely in bone and thus cannot be compared.

With regard to BMAT quantification, we and others previously published on presence of BMAT in similar age animals in long bones, femur and/or tibia (14, 15, 40, 41, 89, 90), as well as validating this method against histology and osmium-µCT (15) (**Supplementary Figure 1, Extended Methods**). High resolution Magnetic Resonance (MR)-based methods are considered gold standard for analysis of adipose depots and body composition across multiple organisms. MR-based methods to look at BMAT were published as early as1990 in the radiology literature (91) and eventually in the early 2000’s in bone and endocrine fields and beyond (92–102). In sum, high resolution MR based methods are widely used for preclinical and clinical applications, for quantification of white adipose stores, body composition analyses, as well as for quantification of BMAT.

Key limitations of this study include the absence of metabolic and caloric intake measurements, the absence of female sex, the number of animals analyzed via histomorphometry, as well as the lack of additional age groups. In addition to quantification of adipose stores noted, it will be clinically meaningful for future studies to quantify mechanical non-marrow depots in the feet and around the tail in SEIPIN KO, as this was shown to differ in clinical syndromes (23, 103). Our team previously published on the metabolic parameters in the same Bscl2 KO mice; our new findings with regard to effects of exercise on bone formation findings suggest that future studies should be initiated to investigate whether running alters metabolic health in the SEIPIN KO condition. Due to the metabolic dysfunction seen in Bscl2, these measurements are highly relevant and may impact bone and BMAT outcomes. SEIPIN deficient mice, including male mice of similar age, have been extensively documented to exhibit metabolic dysfunction consistent with similar clinical dysfunction documented in CGL patients (31, 104).

In sum, our data provide evidence for BMAT as a unique adipose depot in the context of congenital generalized lipodystrophy. Concomitant with global reduction of white adipose stores, marrow adiposity was reduced in SEIPIN KO, particularly in the proximal femur. Cortical bone geometry was negatively altered in KO, while trabecular bone was unaffected. Exercise-induced trabecular bone was possible, despite lack of attenuation in BMAT, highlighting the likelihood that SEIPIN deficient mice rely on alternative substrates to fuel bone anabolism. The lack of prior research on physical exercise in lipodystrophy, whether clinically or mechanistically, represents a significant knowledge gap. Our work demonstrates a beneficial impact of exercise on bone in a mouse model of severe CGL. Future studies are needed to understand the metabolic benefit of exercise for lipodystrophy and how this relates to musculoskeletal health.

## Data Availability Statement

The original contributions presented in the study are included in the article/**Supplementary Material**. Further inquiries can be directed to the corresponding author.

## Ethics Statement

The animal study was reviewed and approved by the University of North Carolina’s Institutional Animal Care and Use Committee (IACUC).

## Author Contributions

MS, JR, and RAC contributed to conception and design of the study. CM, JSS, BS, ZX, XZ, MAS, and ELK collected and/or analyzed data. Both CM and SEL-L organized, analyzed data, and drafted portions of the manuscript. MS wrote the final version of the manuscript. All authors contributed to manuscript revision, read, and approved the submitted version.

## Funding

Grant Support: NIH awards: MS: R01AR073264 and KL2TR002490, JR: R01AR075803, EK: R01DK107481.

## Conflict of Interest

The authors declare that the research was conducted in the absence of any commercial or financial relationships that could be construed as a potential conflict of interest.

## Publisher’s Note

All claims expressed in this article are solely those of the authors and do not necessarily represent those of their affiliated organizations, or those of the publisher, the editors and the reviewers. Any product that may be evaluated in this article, or claim that may be made by its manufacturer, is not guaranteed or endorsed by the publisher.
