# Supplementary material for: Exercise Increases Bone in SEIPIN Deficient Lipodystrophy, Despite Low Marrow Adiposity
Source: Front Endocrinol (Lausanne). 2022 Jan 25;12:782194. doi: 10.3389/fendo.2021.782194 (PMC8822583; doi:10.3389/fendo.2021.782194)
Supplement: Supplementary file 1 [file DataSheet_1.pdf]

Gene	Forward Primer	Reverse Primer
**Perilipin 1**	5'-CCATGTCCCTATCCGATGCC-3'	5'-TGGGAAGCGGCACATAGTGTA-3'
**Perilipin 5**	5'-TCTGATTCTCTGACCGCTGC-3'	5'- ACCGGACATTCTGCTGTGTG-3'
**UCP1**	5'-TGGAAAGGGACGACCCCTAAT-3'	5'-ACAGTAAATGGCAGGGGACG-3'
**CtytC**	5'-GCAAGCATAAGACTGGACCAAA-3'	5'-TTCTTGGCATCTGTGTAAGAGAATC-3'
**APN**	5'-CACCAAAAGGGCTCAGGATGC-3'	5'-AACGTCATCTTCGGCATGACT-3'
**ELOVL3**	5'-TGGACCTGATGCAACCCTATG-3'	5'-GAGCTTACCCAGTACTCCTCC-3'
**SOST**	5'-ATCCCAGGGCTTGGAGAGTA-3'	5'-ATCCCAGGGCTTGGAGAGTA-3'
**SCOT**	5'-GAGGACGGCATGTACGCTAA-3'	5'-GAGGACGGCATGTACGCTAA-3'

Supplementary table 1. Primer sequences.
