# Supplementary material for: Exercise Increases Bone in SEIPIN Deficient Lipodystrophy, Despite Low Marrow Adiposity
Source: Front Endocrinol (Lausanne). 2022 Jan 25;12:782194. doi: 10.3389/fendo.2021.782194 (PMC8822583; doi:10.3389/fendo.2021.782194)
Supplement: Supplementary file 2 [file Presentation_1.pdf]

Expanded Supplemental Methods : 3D Quantification of Bone Marrow Adipose Tissue (BMAT) via 9.4T MRI with Advanced Image Analysis

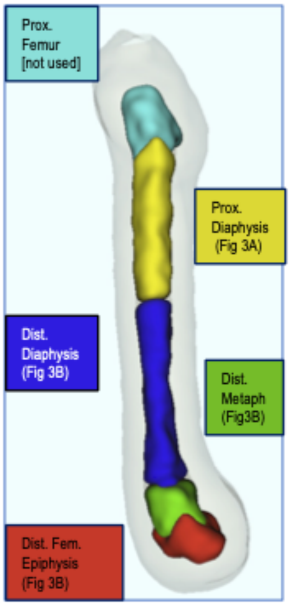

Figure 1 of the Expanded Supplemental Methods. Regional Subdivision for Quantification of BMAT.

Quantification of BMAT was performed via high-resolution 9.4T MRI with advanced image analysis for 3D volumetric BMAT analysis, a method previously validated against both osmium-stained-μCT with advanced image analysis, as well as histomorphometry (1, 2)). This is the only published image processing workflow for BMAT, and whether applied for MRI or osmium- μCT, the processing involves manual contouring of bones as well as relativizing BMAT depots to an individual bone volume measurement. This method was selected because it allows a reproducible, whole bone, 3-dimensional approach, and visualization of superimposed, group images. Although osmium is useful because it binds lipid avidly, osmium stained ex-vivo specimens cannot be compared across batched experiments, and penetration of the osmium can be variable, which may limit its use in studies that are longitudinal or require comparison across multiple groups and time points. Femurs were analyzed with a 9.4T horizontal small-bore MRI scanner to quantify BMAT volumetrically (2). The effect of mechanical loading or weight-bearing exercise on the skeleton in rodents has been shown in hind limbs, e.g., tibia and femur, but also other sites (3-5). Progenitor populations in the femur (6) are more exercise-responsive though both have strong response to mechanical/ exercise intervention and are therefore preferred for murine exercise experiments, as compared with the forelimbs (6). Briefly, water and fat maps were obtained with a 2-dimensional RARE imaging sequence with the following parameters: RARE factor = 4, TE = 28 ms, TR = 4000 ms, number of averages = 4, number of slices = 24, slice thickness = 0.5 mm, in-plane resolution = 100×100 μm2, matrix size = 130×130. Utilizing the fact that the fat and water protons have an NMR frequency separation of 3.5 ppm, a Gaussian-shaped 90-degree saturation pulse with a width of 2 ms was applied preceding the RARE sequence to suppress the fat or water signal while the other signal remained unaffected. Fat and water images were acquired by setting the saturation pulse frequency to be the same as the water and fat frequencies, respectively. For BMAT quantification, we created a regional label map of the femur, excluding cortical bone regions, with regions for the distal epiphysis, distal metaphysis, distal diaphysis, and proximal diaphysis (see Fig 1 of the Expanded Methods). Intensity-weighted volume of BMAT was then quantified via regional fat histograms.

In our processing workflow, we manually subdivided the full images containing all 10 samples into individual images for each bone. Then, we employed the water images to manually outline femoral bone masks using Insight ITK-SNAP (openly available at www.itksnap.org (7). Using these bone masks, interior bone regions were masked from other image parts in both the water and fat maps. Next, we established a common, study-specific reference space by computing an unbiased average image (8) from the masked water maps using the ANTs registration software (9). All individual water and fat maps were then propagated into the common space, where voxel-wise correspondence allows direct comparison of intensities. Average fat maps for each group were computed in the common space and superimposed on the common, average water image for

visualization of group fat maps as in Figs 2A , 3D. Fat map intensities were represented with a colored heat map in 3DSlicer for visualization (7).

ITK-SNAP Open Access Software for Image Analysis

This open access software (openly available at www.itksnap.org (7) provides semi-automatic segmentation via active contour methods, as well as manual delineation and image navigation. Compared to other, larger open-source image analysis tools, ITK-SNAP focuses specifically on the problem of image segmentation, and unrelated features are kept to a minimum. The design emphasizes interaction and ease of use, with the bulk of the development effort dedicated to the user interface. ITK-SNAP 3.2 (2019) was the first major release of ITK-SNAP in several years funded by the NIH.

3D Slicer open-access Platform for Medical Image Analysis

3D Slicer (www.slicer.org) is a platform distributed under a BSD-style open-source license that is broadly compatible with the Open-Source Definition by The Open Source Initiative and contains no restrictions on legal uses of the software). 3D Slicer is an open-access research software platform, allowing researchers to develop and evaluate new methods and distribute them to users (10). Features are available and extensible in Python and C++. Slicer has a built-in Python console and can act as a Jupyter notebook kernel with remote 3D rendering capabilities. The license has been in use since 2005 for the 3D Slicer software package that as of 2021 has been downloaded more than a million times and has been referenced in about 12,000 academic publications (https://www.slicer.org/wiki/Main_Page/SlicerCommunity) according to the 3DSlicer site. It was written in part for an NIH-funded consortium who wanted code contributions to be compatible with ultimate use in real-world medical products.

References of the Expanded Supplemental Methods

1. M. Styner, G. M. Pagnotti, C. McGrath, X. Wu, B. Sen, G. Uzer, Z. Xie, X. Zong, M. A. Styner, C. T. Rubin and J. Rubin: Exercise Decreases Marrow Adipose Tissue Through Β-Oxidation in Obese Running Mice. J Bone Miner Res, 32(8), 1692-1702 (2017) doi:10.1002/jbmr.3159
2. C. McGrath, J. S. Sankaran, N. Misaghian-Xanthos, B. Sen, Z. Xie, M. A. Styner, X. Zong, J. Rubin and M. Styner: Exercise Degrades Bone in Caloric Restriction, Despite Suppression of Marrow Adipose Tissue (MAT). J Bone Miner Res, 35(1), 106-115 (2020) doi:10.1002/jbmr.3872
3. N. M. Ocarino, U. Marubayashi, T. G. Cardoso, C. V. Guimaraes, A. E. Silva, R. C. Torres and R. Serakides: Physical activity in osteopenia treatment improved the mass of bones directly and indirectly submitted to mechanical impact. J Musculoskelet Neuronal Interact, 7(1), 84-93 (2007)
4. L. J. Pereira, S. Macari, C. C. Coimbra, T. Pereira, B. R. Barrioni, R. S. Gomez, T. A. Silva and S. M. Paiva: Aerobic and resistance training improve alveolar bone quality and interferes with bone-remodeling during orthodontic tooth movement in mice. Bone, 138, 115496 (2020) doi:10.1016/j.bone.2020.115496
5. G. M. Pagnotti, M. E. Chan, B. J. Adler, K. R. Shroyer, J. Rubin, S. D. Bain and C. T. Rubin: Low intensity vibration mitigates tumor progression and protects bone quantity and quality in a murine model of myeloma. Bone, 90, 69-79 (2016) doi:10.1016/j.bone.2016.05.014

6. I. J. Wallace, G. M. Pagnotti, J. Rubin-Sigler, M. Naeher, L. E. Copes, S. Judex, C. T. Rubin and B. Demes: Focal enhancement of the skeleton to exercise correlates with responsivity of bone marrow mesenchymal stem cells rather than peak external forces. J Exp Biol, 218(Pt 19), 3002-9 (2015) doi:10.1242/jeb.118729

7. P. A. Yushkevich, J. Piven, H. C. Hazlett, R. G. Smith, S. Ho, J. C. Gee and G. Gerig: User-guided 3D active contour segmentation of anatomical structures: significantly improved efficiency and reliability. Neuroimage, 31(3), 1116-28 (2006) doi:10.1016/j.neuroimage.2006.01.015

8. S. Joshi, B. Davis, M. Jomier and G. Gerig: Unbiased diffeomorphic atlas construction for computational anatomy. Neuroimage, 23 Suppl 1, S151-60 (2004) doi:10.1016/j.neuroimage.2004.07.068

9. B. B. Avants, N. J. Tustison, G. Song, P. A. Cook, A. Klein and J. C. Gee: A reproducible evaluation of ANTs similarity metric performance in brain image registration. Neuroimage, 54(3), 2033-44 (2011) doi:10.1016/j.neuroimage.2010.09.025

10. A. Fedorov, R. Beichel, J. Kalpathy-Cramer, J. Finet, J. C. Fillion-Robin, S. Pujol, C. Bauer, D. Jennings, F. Fennessy, M. Sonka, J. Buatti, S. Aylward, J. V. Miller, S. Pieper and R. Kikinis: 3D Slicer as an image computing platform for the Quantitative Imaging Network. Magnetic Resonance Imaging, 30(9), 1323-41 (2012) doi:10.1016/j.mri.2012.05.001
